# The influence of calcitonin gene-related peptide on markers of bone metabolism in MG-63 osteoblast-like cells co-cultured with THP-1 macrophage-like cells under virtually osteolytic conditions

**DOI:** 10.1186/s12891-016-1044-5

**Published:** 2016-05-04

**Authors:** Heidrun Jablonski, Heike Rekasi, Marcus Jäger

**Affiliations:** University Hospital Essen, Department of Orthopedic and Trauma Surgery, University of Duisburg-Essen, Hufelandstrasse 55, Essen, 45147 Germany

**Keywords:** MG-63 osteoblasts, THP-1 macrophages, Co-culture, Osteolysis, Particles, LPS, Inflammation, Bone metabolism, CGRP

## Abstract

**Background:**

The neuropeptide calcitonin gene-related peptide (CGRP) has been described to have an inhibitory effect on endotoxin- and wear particle-induced inflammation in the early stages of periprosthetic osteolysis. In the present study, the crosstalk between immune cells and osteoblasts in osteolytic conditions treated with CGRP has been analyzed to evaluate whether the anti-inflammatory properties of the peptide also have a beneficial, i.e. an anti-resorptive and osteo-anabolic impact on bone metabolism.

**Methods:**

MG-63 osteoblast-like cells were co-cultured with THP-1 macrophage-like cells stimulated with either ultra-high molecular weight polyethylene (UHMWPE) particles or different concentrations of bacterial lipopolysaccharides (LPS) and simultaneously treated with CGRP. Inflammation was monitored in terms of measuring the levels of tumor necrosis factor (TNF)-α secretion. Furthermore, the production of the osteoblast markers osteoprotegerin (OPG), receptor activator of nuclear factor κB ligand (RANKL), alkaline phosphatase (ALP) and osteopontin (OPN) was quantified. Also, ALP enzymatic activity was measured.

**Results:**

Stimulation of co-cultured THP-1 macrophages with either high levels of LPS or UHMWPE induced the secretion of TNF-α which could be inhibited by CGRP to a great extent. However, no remarkable changes in the OPG/RANKL ratio or bone ALP activity were observed. Interestingly, OPN was exclusively produced by THP-1 cells, thus acting as a marker of inflammation. In addition, TNF-α production in THP-1 single cell cultures was found to be considerably higher than in co-cultured cells.

**Conclusions:**

In the co-culture system used in the present study, no obvious relation between inflammation, its mitigation by CGRP, and the modulation of bone metabolism became evident. Nonetheless, the results suggest that during the onset of periprosthetic osteolysis the focus might lie on the modulation of inflammatory reactions. Possibly, implant-related inflammation might merely have an impact on osteoclast differentiation rather than on the regulation of osteoblast activity.

**Electronic supplementary material:**

The online version of this article (doi:10.1186/s12891-016-1044-5) contains supplementary material, which is available to authorized users.

## Background

Aseptic loosening due to particle-induced osteolysis remains a major complication associated with total joint replacement procedures. Thereby, detrimental effects on bone anchoring the implant are elicited by prosthesis-derived wear debris consisting of various materials such as titanium, polyethylene, and ceramics. Amongst these, ultra-high molecular weight polyethylene (UHMWPE) particles are thought to play a leading role in the development of periprosthetic bone resorption due to their size and biological activity [1, 2]. Furthermore, both soluble and adsorbed endotoxins in subclinical concentrations are described to play a role in the onset of aseptic osteolysis [3, 4]. Both particulate wear debris and endotoxins interact with immune cells in the periprosthetic interface membrane surrounding the implant. Upon contact with or phagocytosis by the cells—primarily macrophages—an inflammatory response is elicited which leads to the secretion of a variety of pro-inflammatory cytokines and chemokines. These in turn promote the differentiation and activation of bone resorbing osteoclasts facilitating osteolysis [5].

At the implant interface bone cells such as osteoclasts and osteoblasts are in close contact to fibroblasts and macrophages of the synovial membrane [6, 7]. Because of this spatial proximity a direct interplay between the immune system and bone seems reasonable. Also, immune cells and bone cells are known to share a number of signaling molecules which link immunological reactions and skeletal metabolism, a connection also termed “osteoimmunology” [8].

Interactions between bone cells and macrophages have been described previously [9]: osteoblasts are able to respond to soluble factors released by macrophages contributing to the modulation of both macrophage and osteoclast activity. Since we have previously shown an inhibitory effect of the neuropeptide calcitonin gene-related peptide (CGRP) on pro-inflammatory cytokine production by macrophages [10] we wondered whether this would directly contribute to an alteration of bone cell biology and bone metabolism. Therefore, we analyzed the interaction of particle- and LPS-stimulated macrophages with bone forming osteoblasts in the present study. The osteoblastic proteins receptor activator of nuclear factor kappa B ligand (RANKL) and osteoprotegerin (OPG) are thought to play a key role in the regulation of bone metabolism whereby the ratio of these proteins determines the rate of bone resorption [11]. Therefore, we questioned whether the osteoblastic production of OPG and RANKL was influenced by pro-inflammatory macrophages and whether CGRP treatment had an effect on the OPG/RANKL ratio. Moreover, the osteoblast activity markers alkaline phosphatase (ALP) and osteopontin (OPN) were analyzed under inflammatory conditions treated with CGRP. Thus, we examined whether the neuropeptide CGRP could implicitly influence osteoblast activity by modulating the immune response of macrophages.

## Methods

### Calcitonin gene-related peptide

Human CGRP (Sigma Aldrich, Saint Louis, Missouri, USA) was dissolved in dimethyl sulfoxide (DMSO; Sigma Aldrich, Saint Louis, Missouri, USA), further diluted in Dulbecco’s phosphate-buffered saline (DPBS; Sigma-Aldrich, Saint Louis, Missouri, USA) and stored at −20 °C until use. During the experiments, THP-1 cells were treated with a commonly used final concentration of 10^−8^ M CGRP often shown to exert maximal effects in cell culture [10, 12–16] while equal amounts of DPBS were added to the corresponding controls.

### Particles

UHMWPE particles (Ceridust VP3610) with a mean particle size (given as equivalent circle diameter) of 1.75 ± 1.43 μm (range 0.06–11.06 μm) were provided by Clariant (Gersthofen, Germany) [17]. For use in cell culture experiments the particles were cleaned in 99 % ethanol for 24 h and dried in a desiccator afterwards. Endotoxin decontamination was confirmed using a limulus amebocyte lysate (LAL) assay (Charles River, Kent, United Kingdom) with a sensitivity of 0.25 EU/ml following the manufacturer’s instructions. Subsequently, the particles were dissolved in sterile 10 % endotoxin-free bovine serum albumin (BSA; Sigma Aldrich, Saint Louis, Missouri, USA) in order to achieve good contact with the cells [18]. Flow cytometry (BD FACSCalibur; BD Biosciences, Heidelberg, Germany) was used to determine the number of particles per volume of solution. For the experiments, UHMWPE particles were added to THP-1 cells at a cell-to-particle ratio of 1:500 which has previously been shown to exert major effects in both inflammatory and bone cells [10, 16, 19, 20].

LPS from *Escherichia coli 055:B6* (Sigma Aldrich, Saint Louis, Missouri, USA) was used as a further inducer of osteolysis-associated inflammation. LPS was reconstituted in DPBS and stored at −20 °C until use. During the experiments, LPS was added to the cells at two different concentrations representing low (10 pg/ml) and high (100 ng/ml) endotoxin levels [21, 22].

### Cells

The acute human monocytic leukemia cell line THP-1 (CLS Cell Lines Service, Eppelheim, Germany) was cultured in RPMI-1640 medium (GE Healthcare, Chalfont St. Giles, United Kingdom) supplemented with 10 % fetal calf serum (FCS; GE Healthcare, Chalfont St. Giles, United Kingdom), 100 U/ml penicillin (Gibco, Darmstadt, Germany), 100 μg/ml streptomycin (Gibco, Darmstadt, Germany) and 2 mM L-glutamine (Gibco, Darmstadt, Germany) in a humidified environment at 5 % CO_2_ and 37 °C. For the experiments, the cells were transferred into 6-well polyethylene terephthalate (PET) transwell permeable supports with a pore size of 0.4 μm (Corning, Acton, Massachusetts, USA) at a quantity of approximately 5.5 × 10^5^ cells per membrane [10]. In order to enhance phagocytic activity, THP-1 monocytes in suspension were differentiated into adherent macrophage-like cells using phorbol-12-myristate-13-acetate (PMA; Calbiochem, Darmstadt, Germany), at a final concentration of 50 nM for 96 h [23–25]. Thereby, the medium was changed once after an initial 72 h of incubation.

The human osteosarcoma cell line MG-63 (CLS Cell Lines Service, Eppelheim, Germany) was used as a model system for osteoblasts [26]. Adherent growing cells were cultured in DMEM/Ham’s F12 medium (Biochrom, Berlin, Germany) supplemented with 10 % FCS (GE Healthcare, Chalfont St. Giles, United Kingdom), 100 U/ml penicillin (Gibco, Darmstadt, Germany), 100 μg/ml streptomycin (Gibco, Darmstadt, Germany) and 2 mM L-glutamine (Gibco, Darmstadt, Germany) in a humidified environment at 5 % CO_2_ and 37 °C. For the experiments, the cells were transferred into 6-well flat-bottomed cell culture plates (BD Biosciences, Heidelberg, Germany) at a quantity of approximately 1 × 10^5^ cells per well [16]. Thereby, about 75 % confluence was reached after 24 h of cell seeding.

### Co-culture

THP-1 cells were differentiated in cell culture inserts for 96 h while MG-63 cells were seeded in 6-well cell culture plates 24 h prior to the experiment and incubated separately as described above. The cells were washed once in DPBS before the inserts containing THP-1 cells were added to the MG-63 cells in order to generate indirect co-cultures. Inserts without THP-1 cells were used as an internal control. RPMI containing LPS, UHMWPE and/or CGRP was added to the inserts (Table 1) while fresh DMEM/Ham’s F12 medium was added to MG-63 cells in the wells. Co-culture of macrophage- and osteoblast-like cells simulating the environment surrounding prostheses during the process of aseptic loosening was performed for 6, 24, and 48 h of incubation. Cell culture media were collected upon termination of the experiments at each time point. Insoluble material was pelleted by centrifugation at 200 × g and 4 °C for 10 min and the supernatants were stored at −20 °C until further use. Furthermore, total RNA was extracted from MG-63 cells after 6 and 24 h of incubation while cell lysates for the determination of osteoblastic ALP activity were generated after 24 and 48 h of incubation.

**Table 1:** MG-63 osteoblasts co-cultured with THP-1 macrophages under virtually osteolytic conditions treated with CGRP

Incubation time	Stimulation				Treatment
	Negative Control	LPS (10 pg/ml)	LPS (100 ng/ml)	UHMWPE (1:500)	
6 h	MG-63 Control	MG-63 Control	MG-63 Control	MG-63 Control	Control
	MG-63 Control	MG-63 Control	MG-63 Control	MG-63 Control	CGRP (10^−8^ M)
	MG-63 + THP-1	MG-63 + THP-1	MG-63 + THP-1	MG-63 + THP-1	Control
	MG-63 + THP-1	MG-63 + THP-1	MG-63 + THP-1	MG-63 + THP-1	CGRP (10^−8^ M)
24 h	MG-63 Control	MG-63 Control	MG-63 Control	MG-63 Control	Control
	MG-63 Control	MG-63 Control	MG-63 Control	MG-63 Control	CGRP (10^−8^ M)
	MG-63 + THP-1	MG-63 + THP-1	MG-63 + THP-1	MG-63 + THP-1	Control
	MG-63 + THP-1	MG-63 + THP-1	MG-63 + THP-1	MG-63 + THP-1	CGRP (10^−8^ M)
48 h	MG-63 Control	MG-63 Control	MG-63 Control	MG-63 Control	Control
	MG-63 Control	MG-63 Control	MG-63 Control	MG-63 Control	CGRP (10^−8^ M)
	MG-63 + THP-1	MG-63 + THP-1	MG-63 + THP-1	MG-63 + THP-1	Control
	MG-63 + THP-1	MG-63 + THP-1	MG-63 + THP-1	MG-63 + THP-1	CGRP (10^−8^ M)

THP-1 macrophage-like cells, co-cultured with MG-63 osteoblast-like cells, were subjected to various virtually osteolytic stimuli (negative control, LPS concentrations of 10 pg/ml and 100 ng/ml, and UHMWPE with a cell-to-particle ratio of 1:500) and neuropeptide treatment (control, CGRP) for 6, 24 and 48 h of incubation

### Cell viability

In order to test for compound-mediated cytotoxicity, both cell types were separately incubated in 96-well flat-bottomed cell culture plates (BD Biosciences, Heidelberg, Germany) together with the various compounds used in the study for up to 48 h. To analyze for cell-mediated cytotoxicity the cells were co-cultured in a 96-well HTS transwell tissue culture system (Corning, Acton, Massachusetts, USA) for up to 48 h. Potential cytotoxic effects were determined by measuring lactate dehydrogenase (LDH) activity in cell culture media using a commercially available LDH assay kit (Pierce Biotechnology, Rockford, Illinois, USA) according to the manufacturer’s specifications.

Compound-affected cell viability was reduced by high levels of LPS and UHMWPE particles as compared to the corresponding negative control at selected time points. However, no remarkable changes were observed with overall viability ranging between 95–100 % for MG-63 and 94–99 % for THP-1 cells in comparison to the untreated control (93–100 % and 98–100 %, respectively). Also, viability was not considerably decreased in co-culture conditions ranging between 97–100 %.

### RNA isolation and quantitative RT-PCR

Total RNA was extracted from co-cultured MG-63 osteoblast-like cells using the NucleoSpin RNA Kit (Macherey-Nagel, Dueren, Germany) according to the manufacturer’s instructions and stored at −70 °C until use. RNA concentration and purity were determined photometrically using the NanoDrop ND-1000 system (Peqlab, Erlangen, Germany). Single-stranded cDNA was synthesized from 500 ng of total RNA at 42 °C for 60 min by reverse transcription using the RevertAid H^−^ First Strand cDNA synthesis kit (Thermo Fisher Scientific, Waltham, Massachusetts, USA) with oligo(dT)_18_ primers. Subsequently, 12.5 ng of cDNA were amplified by quantitative polymerase chain reaction employing the QuantiTect SYBR Green PCR kit (Qiagen, Hilden, Germany) following the manufacturer’s instructions. Amplification of each cDNA sample was performed in duplicate using QuantiTect Primer assays (Qiagen, Hilden, Germany) for the detection of mRNA levels of *RANKL* (fragment size: 91 bp, Cat. No. QT00215614), *OPG* (fragment size: 107 bp, Cat. No. QT00014294), *ALP* (fragment size: 110 bp, Cat. No. QT00012957) and the housekeeping gene glycerine-aldehyde-3-phosphate-dehydrogenase (*GAPDH*; fragment size: 95 bp, Cat. No. QT00079247) using an AB7500 Real-Time PCR Cycler (Applied Biosystems, Darmstadt, Germany). A total number of 45 cycles was performed. For each pair of primers a negative control reaction without cDNA (no template control) was included. To further control for residual genomic DNA contamination, amplification was also performed on samples without reverse transcriptase (no reverse transcription control). The levels of expression of each sample were normalized to the expression of the housekeeping gene *GAPDH*. Results were calculated using the comparative method of relative quantification [27].

### ELISA

Levels of the pro-inflammatory cytokine human TNF-α as well as the osteoblast specific protein secretion of human RANKL, OPG and OPN were quantified in cell culture supernatants using a commercially available enzyme-linked immunosorbent assay (ELISA) kit (R&D Systems, Minneapolis, Minnesota, USA) following the manufacturer’s instructions. All samples were measured in duplicate using the ELx808 microplate reader (BioTek Instruments, Winooski, Vermont, USA) for data acquisition. Protein concentrations were calculated from the appropriate standard curves using the MikroWin 2000 software (Mikrotek Laboratory Systems, Overath, Germany).

### Western blot

MG-63 cells in single cell or co-culture stimulated with either UHMWPE particles or LPS and treated with CGRP for 6–48 h of incubation were collected as described above and total protein was extracted using radioimmunoprecipitation assay (RIPA) buffer (Thermo Fisher Scientific, Waltham, Massachusetts, USA). The cells were lysed for 30 min on ice, sonicated for 30 s at 50 % amplitude using an ultrasonic processor (Type UP100H; Hielscher Ultrasonics, Teltow, Germany) and centrifuged at 13.000 × g for 15 min at 4 °C. The supernatant was recovered and stored at −20 °C until further analysis. Total protein content of the cell lysates was quantified using the Pierce BCA assay kit (Thermo Fisher Scientific, Waltham, Massachusetts, USA) according to the manufacturer’s protocol for the microplate procedure.

Equal amounts of protein (20 μg) along with recombinant human soluble (s)RANKL (100 ng; PeproTech, Hamburg, Germany) and LNCaP whole cell lysate (25 μg, sc-2231; Santa Cruz Biotechnology, Dallas, Texas, USA) were separated by 8–16 % tris-glycine SDS-PAGE (Thermo Fisher Scientific, Waltham, Massachusetts, USA) and then electro-blotted to 0.45 μm nitrocellulose membranes (Bio-Rad, Hercules, California, USA). Following protein transfer, the membranes were blocked with 3 % BSA in PBS containing 0.05 % Tween-20 for 1 h at room temperature before incubation with a rabbit polyclonal anti-human full-length (fl)RANKL antibody (sc-9073, final dilution 1: 200; Santa Cruz Biotechnology, Dallas, Texas, USA) or a rabbit polyclonal anti-human sRANKL antibody (500-P133, final dilution 1:500; PeproTech, Hamburg, Germany) overnight at 4 °C. GAPDH (sc-25778, final dilution 1:1000; Santa Cruz Biotechnology, Dallas, Texas, USA) was used as an internal control. Specifically bound primary antibodies were detected with peroxidase-conjugated secondary antibodies. Protein bands were detected by enhanced chemiluminescence (Pierce ECL Western Blotting Substrate; Thermo Fisher Scientific, Waltham, Massachusetts, USA) and visualized by conventional film-based imaging (GE Healthcare, Chalfont St. Giles, UK).

### ALP activity

Osteoblast specific ALP activity was measured using a colorimetric Alkaline Phosphatase Assay Kit (abcam, Milton, England) according to the manufacturer’s instructions. Upon termination of the experiment MG-63 cells were detached from the cell culture plate, collected at 1000 × g and 4 °C for 5 min and resuspended in 230 μl (24 h) or 350 μl (48 h) of ALP assay buffer. Lysis was performed by five repeated freeze/thaw cycles in liquid nitrogen at −196 °C and a water bath at 37 °C respectively. Cell lysates were stored at −20 °C until use. Prior to measuring the ALP activity of the cell lysates insoluble material was removed by centrifugation at 13.000 × g and 4 °C for 3 min. Aliquots of 80 μl of the supernatants were subjected to analysis in duplicate. The conversion of *para*-nitrophenylphosphate (*p*NPP) chromogenic substrate over 60 min was compared to a standard curve as specified by the manufacturer.

### Statistics

All data are expressed as mean ± standard deviation derived from at least three independent experiments. Statistical analysis was carried out using GraphPad Prism 6 (GraphPad, La Jolla, California, USA). One-way analysis of variance (ANOVA) followed by Tukey post hoc analysis was performed to evaluate differences within and between the experimental groups. Statistical significance was considered at *p* < 0.05 (*). Results were further considered to be very statistically significant (**) at *p* < 0.01 and extremely statistically significant (***) at *p* < 0.001.

## Results

### Both UHMWPE particles and LPS provoke an inflammatory reaction in THP-1 macrophage-like cells

**Fig. 1:**
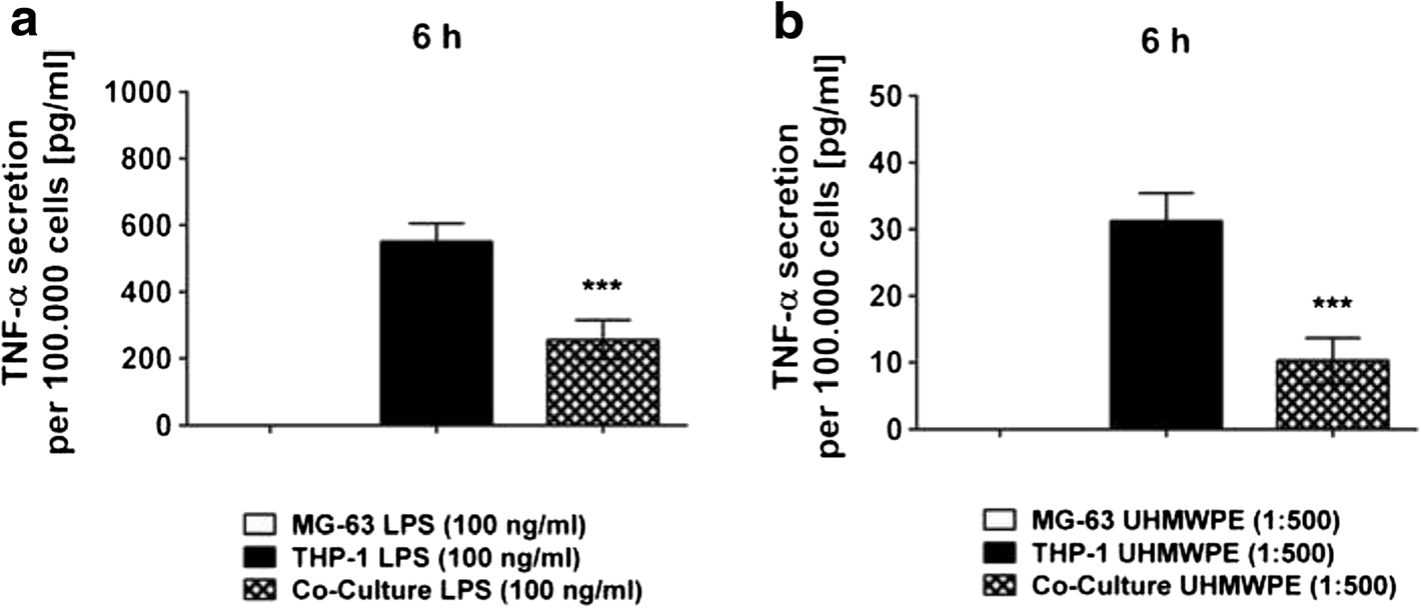
UHMWPE particle- and LPS-induced TNF-α secretion in THP-1 macrophages co-cultured with MG-63 osteoblasts. THP-1 macrophage-like cells co-cultured with MG-63 osteoblasts were stimulated with LPS or UHMWPE particles. The secretion of the pro-inflammatory cytokine TNF-α was quantified in cell culture supernatants. TNF-α secretion [pg/ml] by both co-cultures and controls as per 100.000 cells at 6 h of incubation is exemplarily shown. Absolute TNF-α cytokine levels secreted by activated co-cultures, THP-1 macrophages and MG-63 osteoblasts at 6 h of incubation is illustrated for stimulation with **a** high levels of LPS (100 ng/ml) and **b** UHMWPE particles (cell-to-particle ratio of 1:500). ***p < 0.001

TNF-α secretion upon stimulation of the cells with either UHMWPE particles or LPS could only be detected in co-cultures as compared to osteoblastic cells alone (Fig. 1). This suggested THP-1 macrophages to be the exclusive source of the pro-inflammatory cytokine. As expected, high levels of LPS always induced the strongest cellular response (*p* < 0.001 at 6 h) with TNF-α levels decreasing over time (data for other time points not shown) while UHMWPE induced only minor changes (n.s.) as compared to the control. However, no TNF-α production could be measured using low levels of LPS. Since no pro-inflammatory response which could have further influenced osteoblastic cells in co-culture was evoked by 10 pg/ml of LPS, hereafter only results for high levels of LPS (100 ng/ml) are shown.

In THP-1 cells cultured alone TNF-α was induced by both high LPS concentrations (Fig. 1a) and UHMWPE (Fig. 1b) as described above. Interestingly, secreted TNF-α was much higher at all time points (*p* < 0.001 at 6 h, data for other time points not shown) when compared to cells co-cultured with osteoblasts suggesting that the presence of osteoblastic cells might reduce the inflammatory reaction of THP-1 macrophages in terms of TNF-α production.

### CGRP modulates the inflammatory reaction of THP-1 macrophage-like cells towards wear particles and lipopolysaccharides

**Fig. 2:**
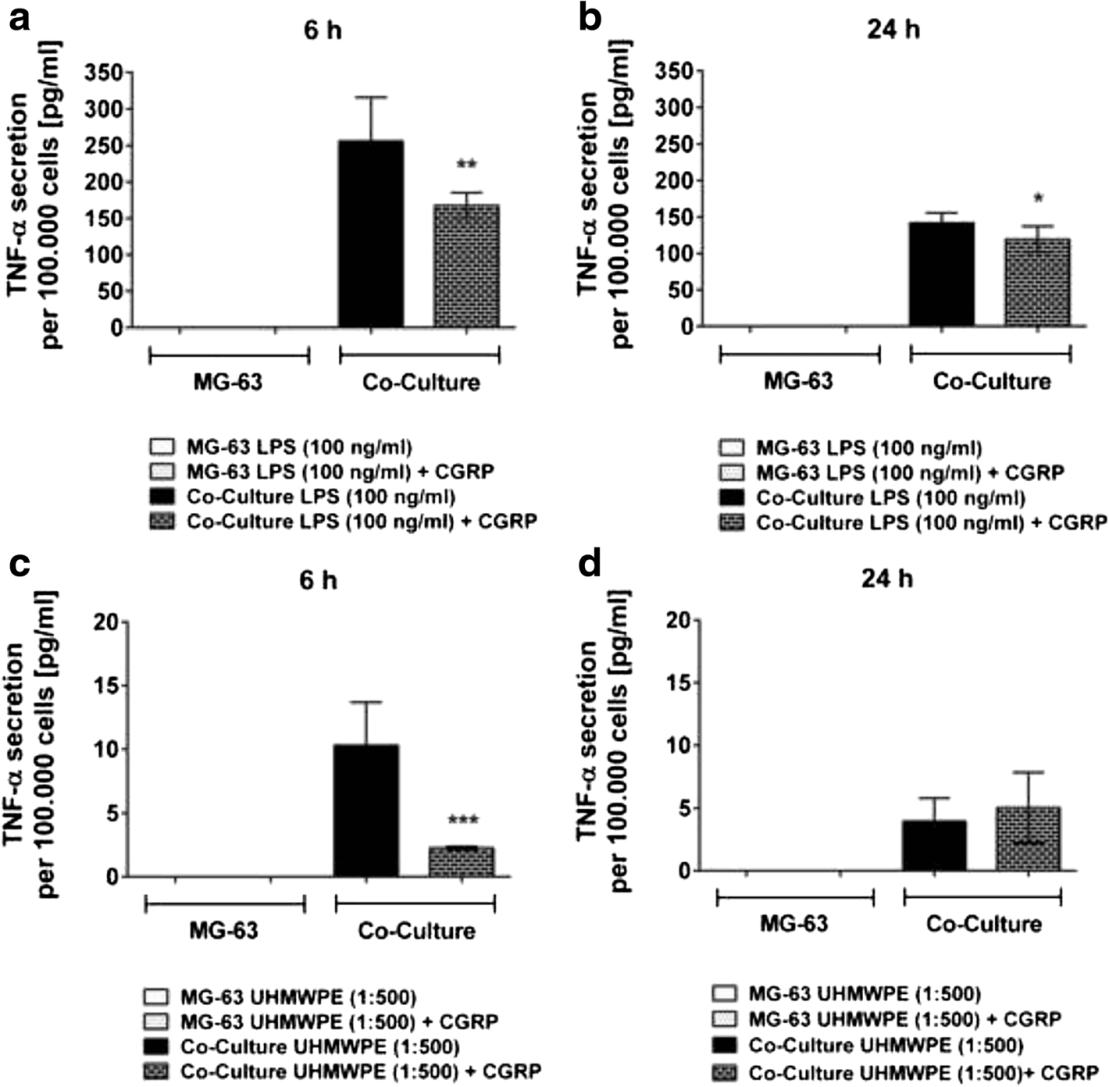
CGRP temporarily inhibits particle- and LPS-induced TNF-α secretion in co-cultured THP-1 macrophages. THP-1 macrophage-like cells co-cultured with MG-63 osteoblasts were stimulated with LPS or UHMWPE particles. The effects of CGRP (10^−8^ M) treatment on co-cultured cells were analyzed. Absolute TNF-α secretion [pg/ml] of co-cultures and controls as per 100.000 cells, activated by high levels of LPS (100 ng/ml) for **a** 6 h and **b** 24 h of incubation or activated by UHMWPE (cell-to-particle ratio of 1:500) for **c** 6 h and **d** 24 h of incubation as compared to the respective TNF-α secretion of activated cells treated with the neuropeptide CGRP. *p < 0.05; **p < 0.01; ***p < 0.001

Both LPS- and particle-induced levels of TNF-α were temporarily inhibited by application of the neuropeptide CGRP in co-cultures of THP-1 and MG-63 cells (Fig. 2). TNF-α secretion as induced by high levels of LPS was mitigated by CGRP at 6 (*p* < 0.01, Fig. 2a) and 24 h (*p* < 0.05, Fig. 2b) of incubation whereas UHMWPE-induced TNF-α was suppressed at 6 h (*p* < 0.001, Fig. 2c) of incubation only (Fig. 2c-d).

### OPG is exclusively produced by osteoblast-like cells

**Fig. 3:**
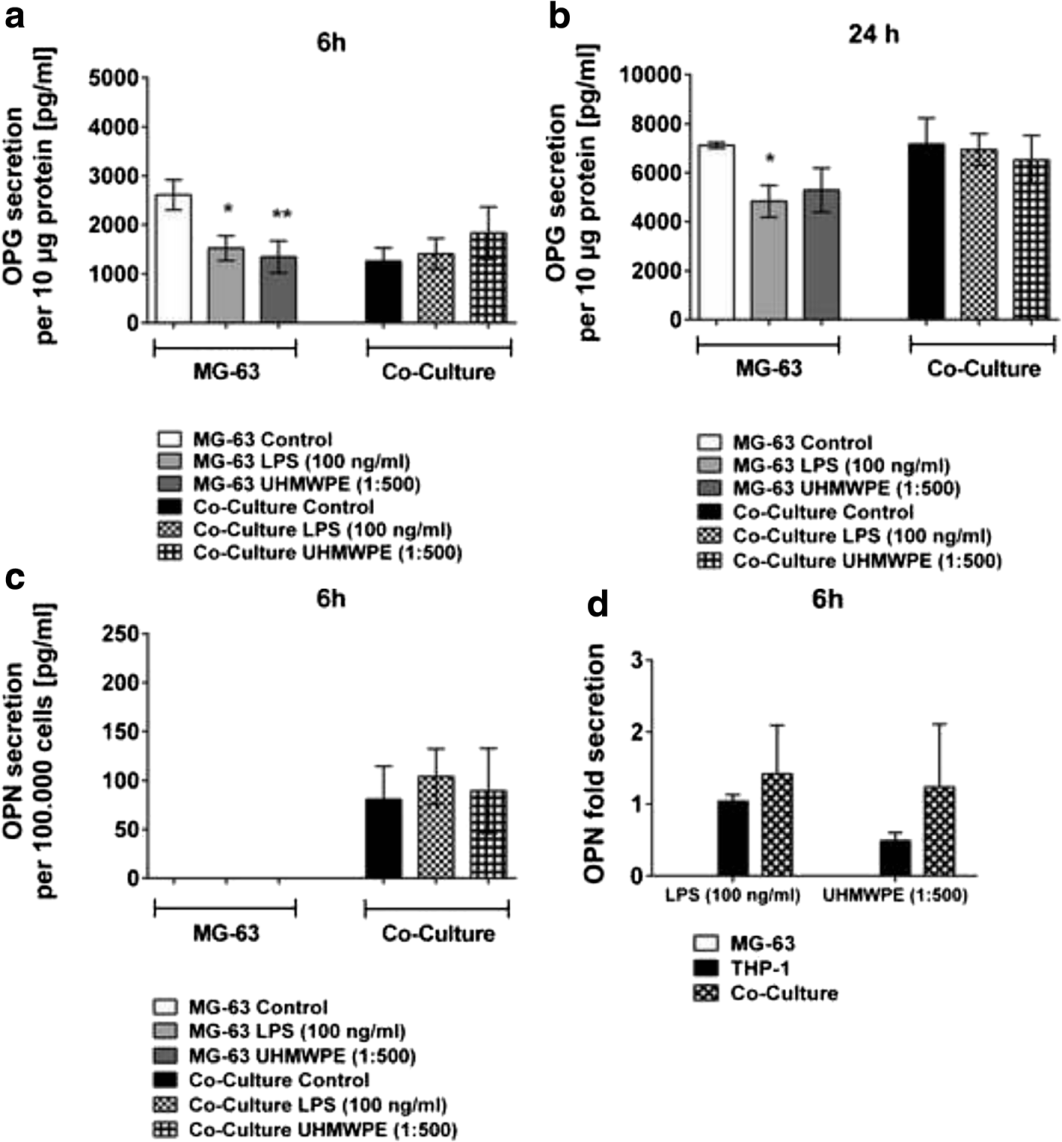
In MG-63 / THP-1 co-cultures OPG, in contrast to OPN, is secreted by osteoblastic cells. THP-1 macrophage-like cells co-cultured with MG-63 osteoblasts were stimulated with LPS (100 ng/ml) or UHMWPE particles (cell-to-particle ratio of 1:500). The secretion of the osteoblastic marker OPG and the multifunctional protein OPN were quantified in cell culture supernatants. OPG secretion [pg/ml] by both co-cultures and controls as per 10 μg of total MG-63 osteoblast-derived protein at 6 h (**a**) and **b** 24 h of incubation is shown. **c** OPN secretion [pg/ml] by both co-cultures and controls as per 100.000 cells at 6 h of incubation is exemplarily shown. **d** OPN secretion, as normalized to the unstimulated control, of MG-63 cells, THP-1 cells and the respective co-cultures activated by LPS (100 ng/ml) or UHMWPE (cell-to-particle ratio of 1:500) is exemplarily shown for 6 h of incubation. THP-1 cells are the source of the protein in the current setup. *p < 0.05; **p < 0.01

The osteoblast specific protein OPG was secreted to a high degree by MG-63 cells exclusively (1126.189 ± 103.301–14491.261 ± 1305.334 pg/ml per 10 μg of total protein in co-cultures and 1035.728 ± 155.053–11.086.096 ± 1536.920 pg/ml per 10 μg of total protein in MG-63 controls while OPG levels in THP-1 single cell cultures where below the detection limit). Secreted OPG levels in osteoblast-like control cultures decreased upon stimulation with both LPS (*p* < 0.05) and UHMWPE particles (*p* < 0.01) whereby significant effects were primarily observed at 6 h of incubation. In contrast, no changes (n.s.) could be observed in co-cultures with THP-1 cells (Fig. 3a-b). The data suggest that, under conditions similar to those in the human body, the focus in the onset of osteolysis might lie on inflammatory reactions rather than on changes in bone metabolism in the first place.

**Fig. 4:**
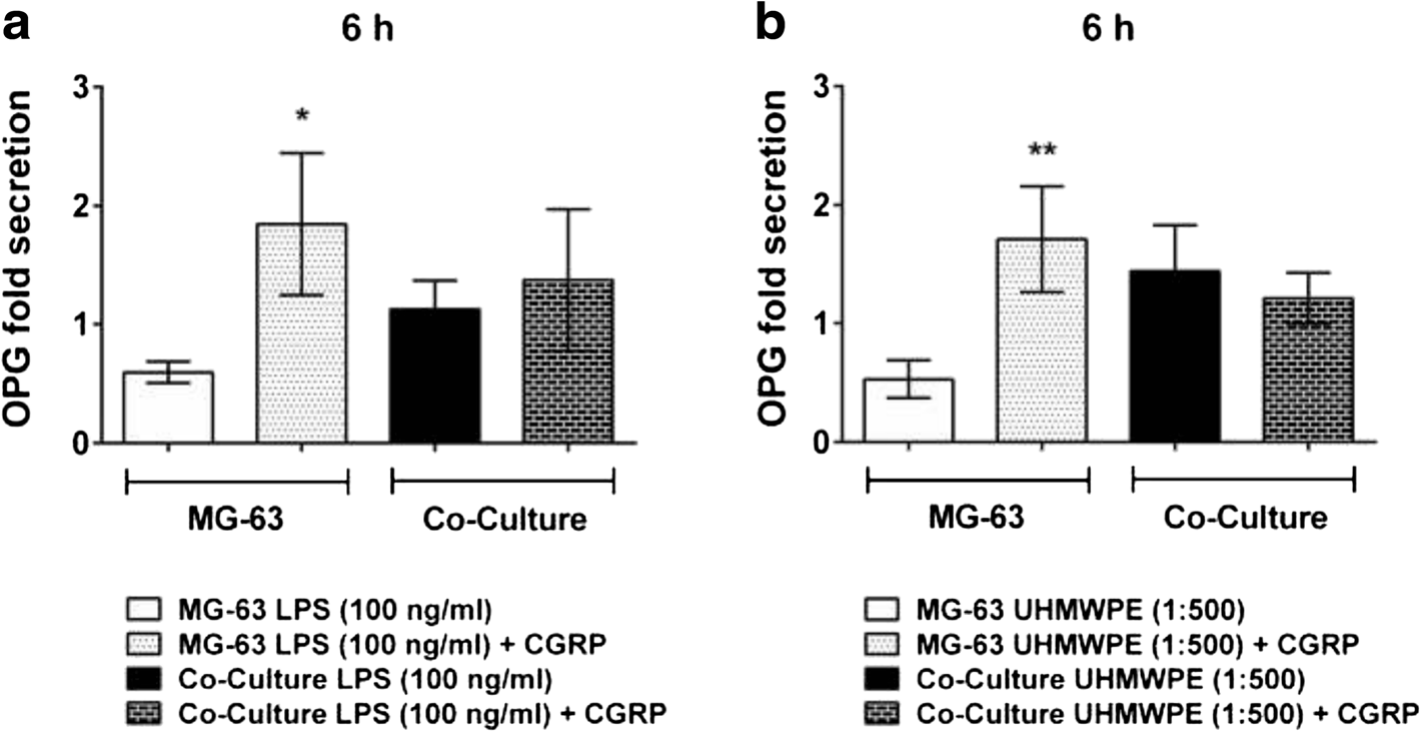
CGRP temporarily augments particle- and LPS-induced OPG secretion in MG-63 osteoblasts cultured alone. THP-1 macrophage-like cells co-cultured with MG-63 osteoblasts were stimulated with LPS or UHMWPE particles. The effects of CGRP (10^−8^ M) treatment on co-cultured cells were analyzed. OPG secretion, as normalized to the unstimulated control, of co-cultures and controls activated by **a** high levels of LPS (100 ng/ml) or **b** UHMWPE (cell-to-particle ratio of 1:500) for 6 h of incubation as compared to the respective OPG fold secretion of activated cells treated with the neuropeptide CGRP. *p < 0.05; **p < 0.01

Remarkably, CGRP could reverse the inhibitory impact of LPS and UHMWPE particles on OPG secretion. However, on the protein level this was observed in MG-63 control cultures only. Thereby, both LPS- (*p* < 0.05, Fig. 4a) and particle- (*p* < 0.01, Fig. 4b) obstructed OPG levels were enhanced by CGRP treatment at 6 h of incubation (Fig. 4, data for other time points not shown). In contrast, only minor and partially adverse effects could be observed in co-cultured cells (n.s.).

### OPN serves as a marker of inflammation rather than of bone mineralization

OPN secretion could only be measured in co-culture supernatants, suggesting its production by THP-1 cells. Indeed, follow up experiments proved that these cells are the exclusive source of OPN in the present experimental setup (Fig. 3d, 6 h, data for other time points not shown). The expression in THP-1 cells cultured alone was not statistically different from co-cultures.

Stimulation of the co-cultured cells with either LPS or UHMWPE particles mostly did not induce significant changes in OPN secretion as compared to the control (6 h, Fig. 3c). However, overall OPN production increased with advancing incubation times (data for other time points not shown). All in all OPN was produced in patterns roughly similar to TNF-α indicating that it might rather be a marker of inflammation than of bone cell activity and mineralization.

**Fig. 5:**
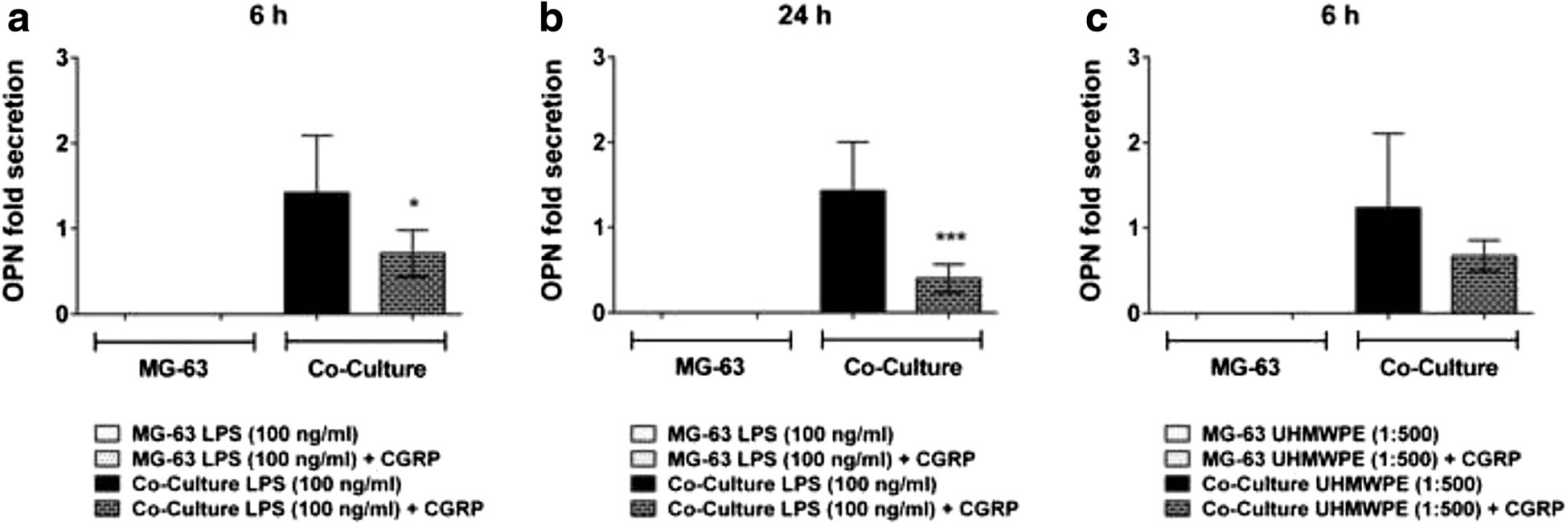
CGRP inhibits THP-1 mediated OPN secretion as elicited by high levels of LPS only. THP-1 macrophage-like cells co-cultured with MG-63 osteoblasts were stimulated with LPS or UHMWPE particles. The effects of CGRP (10^−8^ M) treatment on co-cultured cells were analyzed. OPN secretion, as normalized to the unstimulated control, of co-cultures and controls activated by high levels of LPS (100 ng/ml) for **a** 6 h and **b** 24 h of incubation or activated by **c** UHMWPE (cell-to-particle ratio of 1:500) for 6 h of incubation as compared to the respective OPN fold secretion of activated cells treated with the neuropeptide CGRP. *p < 0.05; **p < 0.01; ***p < 0.001

Intriguingly, CGRP treatment could reduce OPN levels as induced by stimulation with high levels of LPS at 6 (*p* < 0.05, Fig. 5a) and 24 h (*p* = 0.001, Fig. 5b) of incubation. Upon stimulation with UHMWPE (6 h, Fig. 5c) diverse effects could be observed. However, in case of an alleviation of OPN production (6 h) the action of CGRP was not significant (data for other time points not shown).

### MG-63 osteoblastic cells do not produce soluble RANKL during inflammatory reactions

**Fig. 6:**
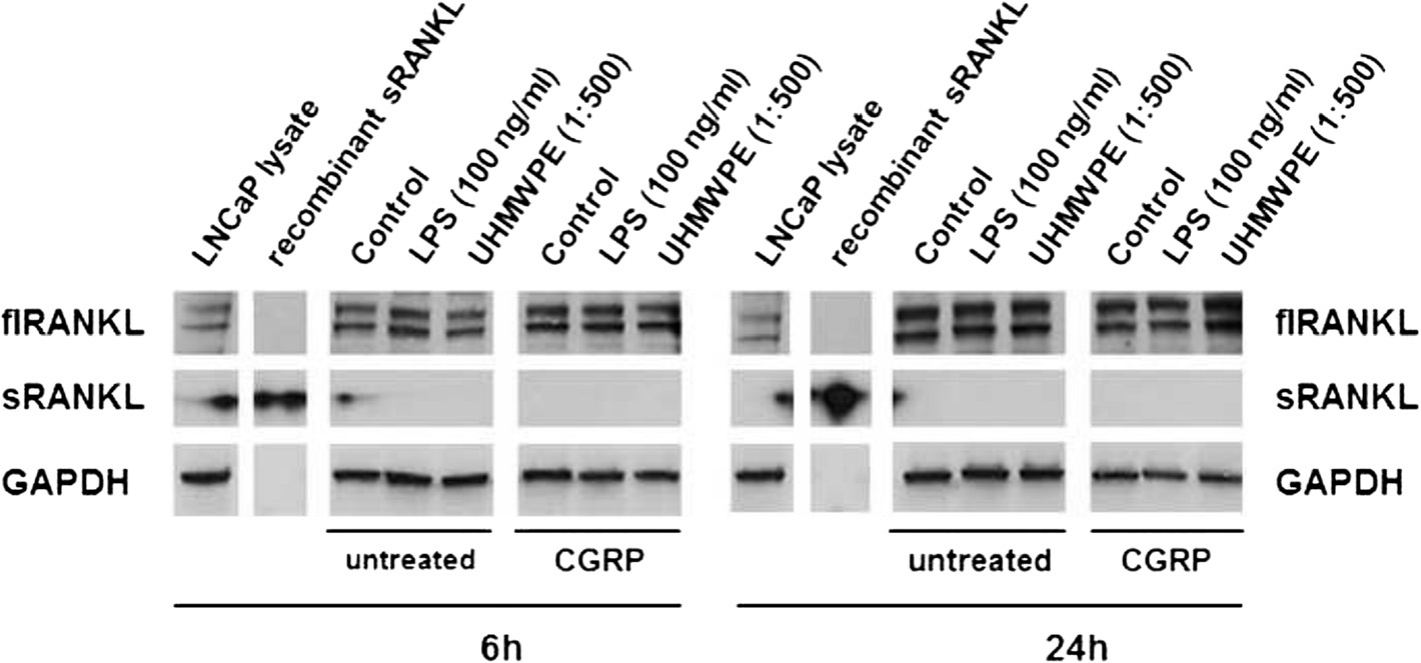
RANKL protein expression in particle- and LPS-stimulated MG-63 osteoblasts remains unchanged even upon CGRP treatment. MG-63 osteoblasts were stimulated with LPS (100 ng/ml) or UHMWPE particles (cell-to-particle ratio of 1:500) and treated with CGRP (10^−8^ M). Changes in the expression of both full-length (fl) and soluble (s)RANKL protein were assessed by SDS-PAGE and Western Blot. RANKL protein expression as compared to commercially available LNCaP lysate and recombinant sRANKL as external controls and GAPDH as an internal control is exemplarily shown for cells at 6 and 24 h of incubation. The sRANKL band provided a very strong signal even upon low exposition times so that artifacts can be seen in the neighboring lanes

Very low mRNA levels of RANKL were measured indicating nearly no expression of the gene (data not shown). Accordingly, no detectable levels of soluble (s)RANKL were produced by either co-cultures or MG-63 osteoblastic cells alone upon stimulation with UHMWPE particles or LPS. In contrast, membrane bound full-length (fl)RANKL was detectable in lysates of activated MG-63 cells using western blotting technique (Fig. 6, data shown for 6 and 24 h of incubation). However, neither stimulation of the cells with UHMWPE and LPS nor treatment with CGRP could induce changes in the expression of the protein.

### ALP activity is hardly influenced by osteolytic conditions and their treatment with CGRP

**Fig. 7:**
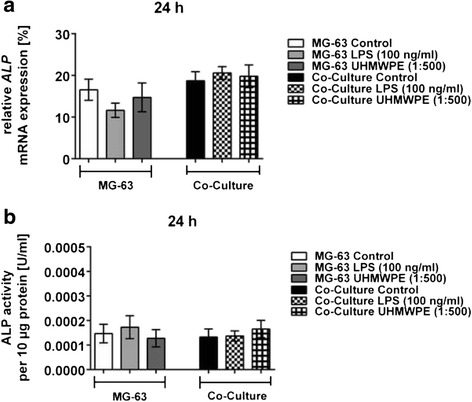
ALP expression and activity are marginally influenced by UHMWPE particles and LPS in co-cultured osteoblasts. THP-1 macrophage-like cells co-cultured with MG-63 osteoblasts were stimulated with LPS (100 ng/ml) or UHMWPE particles (cell-to-particle ratio of 1:500). **a** Relative *ALP* mRNA expression by both co-cultures and controls as compared to the housekeeping gene *GAPDH* at 24 h of incubation is exemplarily shown. **b** ALP activity [U/ml] in both co-cultures and controls as per 10 μg of total protein at 24 h of incubation is exemplarily shown

Bone specific ALP expression levels were hardly affected by stimulation with either LPS or UHMWPE particles (Fig. 7a). Therefore, application of CGRP did not have major effects on the expression of ALP (data not shown).

Correspondingly, ALP activity in MG-63 cell lysates was rather low ranging between 0.00009 and 0.00018 U/ml per 10 μg of total protein (Fig. 7b). Conversion of *p*NPP hardly changed upon stimulation of co-cultures or controls with either LPS or UHMWPE (n.s.). Application of CGRP also did not induce relevant changes of ALP activity (n.s., data not shown).

## Discussion

To the best of our knowledge this is the first report analyzing the influence of the neuropeptide CGRP on co-cultured osteoblasts and macrophages. The present study analyzed whether the previously described anti-inflammatory impact of CGRP on wear particle- and LPS-induced cytokine secretion [10] would have a favorable effect on bone metabolism. It is well known that macrophages play a key role in the regulation of bone remodeling and homeostasis, mainly by secreting cytokines [28]. Thereby, macrophages do not only regulate osteoclast activity and contribute to bone resorption but they also control osteoblast mineralization [29–32]. Thus, we questioned whether the inhibition of the production of pro-inflammatory cytokines by macrophage-like cells upon CGRP treatment would have an influence on markers of bone mineralization such as ALP and OPN or on the production of the osteoblastic proteins OPG and RANKL.

As described earlier for THP-1 macrophages cultured alone [10], also in cells co-cultured with MG-63 osteoblasts both particle- and LPS-induced TNF-α secretion was temporarily inhibited upon treatment with CGRP. Interestingly, in co-cultures TNF-α was secreted to a lesser extent than in macrophage-like cells alone. This suggests that the presence of osteoblasts in the co-culture system might regulate macrophage behavior upon stimulation with either UHMWPE particles or LPS. This would concur with earlier studies reporting on a modulation of the macrophage response by osteoblasts [33]. Indeed, osteoblasts might be able to inhibit primary inflammatory reactions, particularly in response to wear debris, since decreased levels of pro-inflammatory cytokines have been observed in co-culture systems [28, 34]. This anti-inflammatory effect is supposed to be potentially mediated by macrophage-derived prostaglandin E_2_ (PGE_2_) or endogenously produced lipoxin [34, 35].

However, not only macrophage behavior is altered. Previous studies have also shown that macrophage-like cells are able to influence osteoblast behavior in co-culture. For instance, the number, activity, and adhesion of osteoblasts have been described to be decreased in the presence of macrophages [28, 36]. Furthermore, macrophages have been shown to enhance the osteoblast response to wear debris, e.g. with respect to the production of pro-inflammatory cytokines such as IL-6 or GM-CSF [34]. On the other hand, it has been found that monocytes and macrophages are capable of producing the osteoinductive protein bone morphogenetic protein (BMP)-2 which exerts an anabolic effect on osteoblast differentiation and proliferation [30]. However, such osteogenic properties could not be confirmed in the present study since ALP levels as a marker of bone cell activity and mineralization remained unchanged during inflammatory reactions and even upon their treatment with CGRP.

Intriguingly, OPN which has originally been described to be produced by osteoblasts and their precursors and to play an important role in the mineralization and resorption of bone [37–40] was found to be produced in patterns similar to TNF-α in the present study. Although protein levels were not distinctly upregulated upon cellular stimulation, this suggests that in the present study OPN might serve as an indicator of inflammation rather than as a mineralization marker. Indeed, OPN has been described as a rather late differentiation marker previously [41]. Additionally, increased OPN concentrations have been found to be associated with sites of monocyte or macrophage accumulation indicating these cells to be the source of the protein [42]. Actually, we could prove this to be the case by identifying THP-1 cells as the producers of secreted OPN in the setup used here. Although OPN could already be detected in unstimulated cells, which might be a side effect of PMA treatment [43], it even temporarily increased upon stimulation with either LPS or wear particles. This observation is in line with other reports revealing OPN to be involved in inflammation, macrophage recruitment and bone resorption whereby it is particularly produced in response to inflammatory stimuli and pro-inflammatory cytokines [44, 45]. Possibly, OPN and pro-inflammatory cytokines might even stimulate each other’s production to eventually trigger chronic inflammation. This in turn indicates a central role for OPN in osteoclast activation and in wear debris-induced osteolysis [46].

However, it has been shown that the production of the osteoblast-specific proteins OPG and RANKL was not strongly influenced by an inflammatory environment created by THP-1 macrophages. In contrast, the expression of both RANKL and OPG has been described to be dramatically affected by LPS or UHMWPE upon direct contact in osteoblasts cultured alone [16, 20]. Although our data revealed a temporary decrease of OPG in osteoblastic cells upon indirect contact with both LPS and UHMWPE, RANKL production, unlike previously reported, was not changed following cellular stimulation. This observation rather matches previous reports revealing no change in RANKL and OPG levels in co-cultures of osteoblasts and macrophages exposed to wear particles [33]. Also, the application of CGRP had only little effect on the OPG/RANKL ratio. In fact, OPG protein levels were merely temporarily upregulated upon treatment with CGRP while RANKL production remained unchanged which eventually might have a slightly beneficial impact on net bone mass.

Taken together, the results of the present study suggest that in the early stages of periprosthetic osteolysis regulation of inflammation rather than a modulation of bone metabolism is the center of disease pathology. Also, a negligible impact of inflammatory reactions on osteoblast biology is suggested: potentially, the modulation of primary inflammatory reactions to both wear particles and endotoxins might have a stronger impact on osteoclastogenesis instead. As pro-inflammatory cytokines, especially TNF-α, already have been reported to have a strong impact on osteoclast differentiation [47, 48] this should be investigated in appropriate culture systems in the future.

Due to the use of immortalized cell lines the present study is subject to certain limitations. For instance, the unexpected lack of OPN production in MG-63 cells is not observed with primary human osteoblasts (preliminary data, 262.483 ± 9.079–1948.312 ± 20.885 pg/ml depending on time and stimulation). Furthermore, co-culture was performed in a transwell system avoiding cell-to-cell contact. However, a direct contact might be required to be able to detect physiological changes since differences between co-cultures in direct as compared to indirect contact have been observed previously [49]. Unfortunately, due to the differentiation process required for THP-1 macrophages direct co-culture of the cells would seem quite difficult. Even if the THP-1 cells were seeded in the lower compartment, MG-63 cells could only be added after withdrawal of PMA in order to avoid any potential adverse effects on the system. Then again, the previously described spontaneous dedifferentiation of THP-1 macrophages during PMA-withdrawal [50] might limit the duration of experimental observation in this co-culture system. Therefore, to allow for longer-term observations, co-cultures employing primary osteoblasts and monocyte-derived macrophages should be used and are currently being established. Also, these cells more closely resemble tissue-residing cells in the periprosthetic environment. Additionally, the present report lacks a full analysis of the influence of the impact of macrophages on bone metabolism since osteoclasts have not been examined. In the future, culture systems employing specific antibodies against key cytokines involved in periprosthetic osteolysis or co-cultures of macrophages and osteoclast precursors should be used to gain an insight into the impact of wear particle- and endotoxin- mediated inflammation on osteoclast differentiation. Thus, a more detailed understanding of the connection and putative causality between inflammation and bone metabolism can be achieved.

## Conclusions

In the present study, the crosstalk between macrophage-like cells and osteoblastic cells under simulated osteolytic conditions and their treatment with CGRP was analyzed to find out whether the anti-inflammatory properties of the neuropeptide also have a beneficial impact on bone metabolism in terms of osteoblast biology. Although TNF-α secretion by co-cultured cells could be inhibited by CGRP, no remarkable and consistent changes in the OPG/RANKL ratio or bone ALP activity were observed. Interestingly, OPN was found to be exclusively produced by THP-1 macrophages serving as an indicator of inflammation. This suggests that in the initial course of periprosthetic osteolysis focus is on the regulation of inflammation rather than on the modulation of bone metabolism. Nonetheless, a regulatory impact of osteoblasts on macrophages could be confirmed given an attenuated production of TNF-α in co-cultured cells as compared to THP-1 macrophages alone.

## Availability of data and materials

The datasets supporting the conclusions of this article are included within the article and its additional files (Additional files 1, 2 and 3).

## Additional files

Additional file 1: Data and statistical analysis of particle- or LPS-activated THP-1/MG-63 co-cultures. (XLSX 20 kb) Additional file 2: Data and statistical analysis of particle- or LPS-activated THP-1/MG-63 co-cultures treated with the neuropeptide CGRP. (XLSX 19 kb) Additional file 3: Data and statistical analysis of various particle- or LPS-activated cell cultures. (XLSX 16 kb)

## Abbreviations

ALP: alkaline phosphatase
CGRP: calcitonin gene-related peptide
flRANKL: full-length RANKL
LPS: lipopolysaccharide
OPG: osteoprotegerin
OPN: osteopontin
RANKL: receptor activator of nuclear factor kappa B ligand
sRANKL: soluble RANKL
TNF: tumor necrosis factor
UHMWPE: ultra-high molecular weight polyethylene
